# The interaction between Environmental Enrichment and fluoxetine in inhibiting sucrose-seeking renewal in mice depend on social living condition

**DOI:** 10.1007/s00213-022-06124-6

**Published:** 2022-03-30

**Authors:** N. Pintori, A. Piva, V. Guardiani, C. M. Marzo, I. Decimo, C. Chiamulera

**Affiliations:** 1 Section of Pharmacology, Department of Diagnostic and Public Health, Policlinico ‘GB Rossi’, P.le Scuro 10, University of Verona, 37134 Verona, Italy; 2 Department of Biomedical Sciences, Cittadella Universitaria Di Monserrato, University of Cagliari, S.P.8 km 0, 700-09042 Monserrato, Cagliari Italy; 3 Department of Biotechnology, University of Verona, Verona, Italy

**Keywords:** Environmental Enrichment, Fluoxetine, Conditioning, Sucrose, Mice, Renewal, Seeking behaviours, Living environment

## Abstract

**Rationale:**

Several single or combined therapeutic approaches have been developed to treat addiction, however with partial efficacy in preventing relapse. Recently, the living environment has been suggested as a critical intervening factor determining the treatment outcomes. Despite accumulating evidence confirming a role of living conditions in the vulnerability to addictive behaviours, their impact on single or integrative therapeutic strategies preventing relapse is yet to be identified.

**Objectives:**

Here, we explore the possible interaction between brief Environmental Enrichment (EE) exposure and acute fluoxetine administration in inhibiting sucrose-seeking behaviours, and whether this effect could be affected by living environment.

**Methods:**

Social and isolated adult male C57BL/6 mice were trained to sucrose self-administration associated to a specific conditioning context (CxA), followed by a 7-day extinction in a different context (CxB). Afterwards, mice were exposed for 22 h to EE and then injected with fluoxetine (10 mg/kg, i.p.) 1 h before a CxA-induced sucrose-seeking test.

**Results:**

Brief EE exposure and acute fluoxetine administration alone inhibited context-induced sucrose-seeking in both housing conditions; however, they exhibited additive properties only in social condition.

**Conclusions:**

Our data show that social environment may influence the EE/fluoxetine interaction in inhibiting relapse to sucrose. These findings suggest that setting up proper living conditions to boost the efficacy of therapeutic approaches may represent a fundamental strategy to treat addiction disorders.

## Introduction

Addiction is a complex mental disorder characterized by compulsive substance seeking or continued use despite harmful consequences (social, psychological and/or physical) and long-lasting changes in the brain (DSM-5 2013; NIDA 2020). Nowadays, the term addiction does not only refer to dependence on exogenous psychotropic substances, such as cocaine or nicotine, but also to maladaptive behaviours that individuals perform for non-drug rewards despite negative consequences (Marks 1990). These *behavioural addictions* include disorders like gambling, internet, as well as eating addiction (especially for high palatable food, e.g. sucrose). A key issue of addiction is relapse to substance taking after long period of abstinence, as confirmed by the high rates of relapse even after many years (Conklin 2006; Conklin and Tiffany 2002; McLellan et al. 2000). The maladaptive persistence of responding has been extensively demonstrated in animal models of both drugs and sucrose-seeking (see review (Venniro et al. 2020), confirming that energy-dense palatable foods and drugs of abuse can impact similarly the reward brain circuits (Small et al. 2001; Volkow and Morales 2015; Volkow et al. 2012). The vulnerability to addiction and relapse results from the complex interaction between rewarding stimulus exposure, biological factors (genetics, epigenetics, synaptic and neuronal plasticity) and environmental factors (socioeconomic conditions, family and peer relationship, stress, exposure to alternative reinforcers) (Kreek et al. 2005; Piazza and Le Moal 1996; Volkow and Boyle 2018). Indeed, it is well known that the conditioning spatial context (Cx) plays an important role in addiction, promoting reward taking and relapse (Crombag et al. 2008; Crombag and Shaham 2002; Khoo et al. 2017).

Several therapeutic approaches (i.e., pharmacological, behavioural and psychosocial) have been developed to treat addiction, however with partial efficacy in preventing relapse (Balter et al. 2014; Benowitz 2008; Connor et al. 2016; Gupta 2015; Negus and Henningfield 2015). Besides pharmacological interventions, preclinical evidence demonstrated the curative effects of Environmental Enrichment (EE), which is able to reduce addictive behaviours such as drug/food-seeking and -relapse (Grimm et al. 2016, 2008; Solinas et al. 2008, 2010). However, the high number of EE components and factors (e.g. length of exposure, location, type of stimulation, etc.) limits translation into the clinical practice. In order to develop EE protocols with higher translational value and feasibility, researchers have been focusing on two possible strategies: short-term EE exposure and development of ‘enviromimetics’, i.e. molecules that mimic the mechanisms and the potential effects of EE, such as increasing brain-derived neurotrophic factor (BDNF) secretion, glutamatergic or endocannabinoid transmission (Kelly and Hannan 2019; Solinas et al. 2021). Both strategies showed controversial results: for instance, brief EE exposure in rodents attenuates cue-induced sucrose-seeking (Grimm et al. 2016, 2008, 2013; Margetts-Smith et al. 2021; Slaker et al. 2016), whereas it potentiates conditioned context-induced sucrose-seeking (phenomenon called *renewal* or *context-induced reinstatement*) (Pintori et al. 2022). Likewise, enviromimetics such as fluoxetine (selective serotonin re-uptake inhibitor; SSRI) and D-cycloserine (NMDA glutamatergic receptor partial agonist) reduce drug-seeking in animal models (Burmeister et al. 2003; Carroll et al. 1990; Leslie and Norwood 2013; Simon O’Brien et al. 2011; Torregrossa et al. 2010), while fail to reduce relapse in humans (Balter et al. 2014; Benowitz 2008). The apparently clinical failure of SSRIs may be related to the dosage, the treatment duration and the compliance necessary to withdrawal period. Indeed, clinical efficacy in decreasing relapse has been observed with high doses of SSRIs (Covi et al. 1995), such as those used successfully to treat obsessive–compulsive disorders. Consistently, due to its ability in reducing impulsive and compulsive behaviours, FDA has recently approved fluoxetine for treating binge-eating disorders. Importantly, reduction of drug-seeking and -taking has been observed in rats acutely exposed to high dose of fluoxetine and citalopram (10 mg/kg) (Burmeister et al. 2003; Simon O’Brien et al. 2011). Therefore, similar to EE, an increase of clinical efficacy of SSRIs in treating addictive disorders could be obtained by setting up precise treatment’s features.

Currently, a multidisciplinary-integrative approach (pharmacological, environmental and psychosocial) is under investigation for treating addiction and to maintain recovery (Kelly and Daley 2013), even though no preclinical standardized studies evaluated the efficacy of different combinations and related synergic effects. A critical factor determining the therapeutic efficacy, especially of pharmacological treatments, is the living environment (i.e., family, home, work and social environments). The relevant role of living environment on treatment outcome has been extensively demonstrated for depression (see review (Branchi and Giuliani 2021)) and, recently, in a few studies on addiction (Liu et al. 2019; Polcin et al. 2010; Solinas et al. 2010). Clinical and preclinical evidence showed that SSRIs antidepressant efficacy is positively modulated by the quality of the living environment (low vs. high social economic status in humans, stressed vs. enriched condition in mice) (Branchi et al. 2013; Chiarotti et al. 2017; Viglione et al. 2019). For instance, it has been demonstrated that the antidepressant effects of fluoxetine treatment are more pronounced in unpredictable chronic mild stressed rats living in an ethological enriched environment (called *PhenoWorld*) than in standard cages (Castelhano-Carlos et al. 2014). Despite several studies confirmed a role of living conditions in the vulnerability to addiction and relapse (Ajonijebu et al. 2017; Caprioli et al. 2007), there is a lack of literature about their impact on the therapeutic efficacy of either single (e.g. pharmacological) or multidisciplinary-integrative approach to prevent relapse.

In the present paper, we aim to investigate (i) whether brief EE exposure and acute fluoxetine administration alone inhibit context-induced sucrose relapse in mice, (ii) whether they are able to positively interact and, lastly, (iii) whether this effect could be further affected by the living environment. To this end, social and isolated adult mice were trained to sucrose self-administration (S/A) in a specific conditioning context, followed by a 7-day extinction phase in a different context. Afterwards, mice were exposed for 22 h to EE (brief EE) and then injected with fluoxetine (10 mg/kg, i.p.) 1 h before a context-induced sucrose-seeking renewal test.

## Materials and methods

### Animals

Adult male C57BL/6 (approximately 8 weeks of age at the start of experiments, total *n* = 79) (Envigo, Italy) were housed in groups of 6 or 7 per cage (social housing condition, experiment 1) or individually (isolation housing condition, experiment 2) in temperature and humidity-controlled environment (19–23 °C, 60 ± 20%) on a 12-h light/dark cycle, with light ON at 7:30 pm. For the experiment 2 (isolation condition), mice were single housed from 10 days before the start of the sucrose S/A. All mice were food restricted to achieve a reduction of 85% of their baseline weight (daily checked), and food was made available after each experimental session, while water was given ad libitum except during experimental sessions. Animals were trained or tested once daily during the dark phase of the light/dark cycle. All animal care and experimental procedures are reported in compliance with the European Union regulations and the Directive 2010/63/EU and were approved by the ethical committee (OPBA) of the University of Verona and by the Ministry of Health (authorization n. 627/2019-PR).

### Drugs

Fluoxetine (fluoxetine hydrochloride, Sigma-Aldrich, USA) (10 mg/kg) or vehicle solution was administered once in each experiment. Fluoxetine was freshly dissolved in 1% EtOH and 99% saline. All injections were administered intraperitoneally (i.p.) in volumes of 10 ml per 1-kg body weight. Dosing and timing were based on the literature showing that 10 mg/kg of fluoxetine was effective to attenuate cocaine-seeking behaviours (Burmeister et al. 2003) and to block ethanol self-administration (Simon O’Brien et al. 2011) in rats.

### Experimental design timeline

**Fig. 1:**
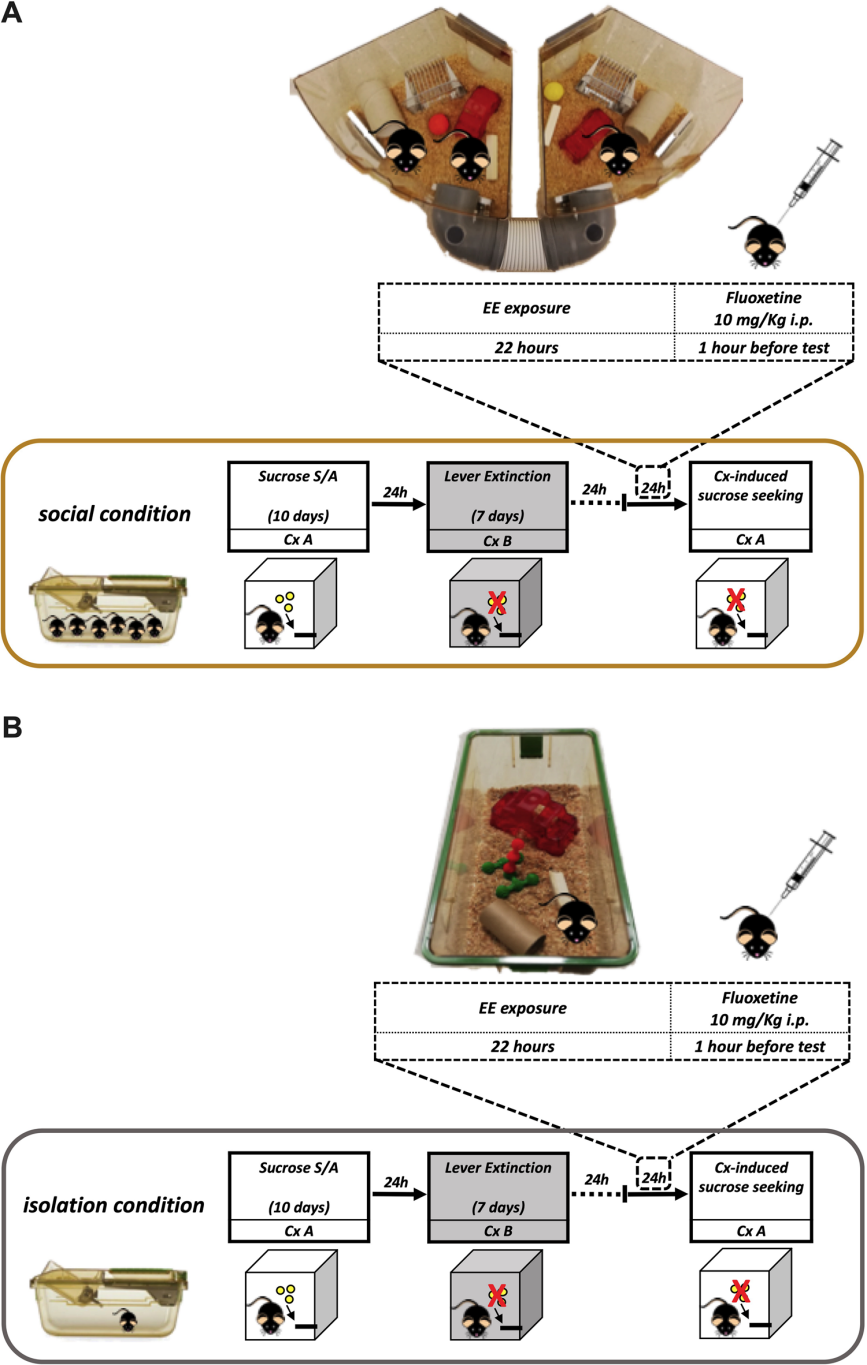
Experimental design timeline. Schematic timeline for experiment 1 **(A)** and 2 **(B)**

Protocol was designed according to Pintori et al. (2022) and Piva et al. (2020) and adapted for the study of renewal in mice (Fig. 1).

Briefly, either social (experiment 1, Fig. 1A) or isolated (experiment 2, Fig. 1B) groups of mice, after a 10-day period of acclimatation, were trained to sucrose S/A associated to a specific conditioning context (CxA, sucrose-paired context), followed by a 7-day extinction phase in a different context (CxB, extinction-paired context). Afterwards, mice were exposed for 22 h to EE and then injected intraperitoneally with fluoxetine 10 mg/kg or vehicle 1 h before CxA-induced sucrose-seeking test (i.e., immediately after the end of EE exposure). Animals were randomly assigned to Exp. 1 and 2 (social and isolation housing conditions) and then to the different treatment conditions (NoEE, EE, Fluoxetine, EE-Fluoxetine, *n* = 10 per group), counterbalancing the subdivision of mice in order to exclude possible lever responding bias. The experimental group sizes (*n* ≥ 8) were chosen based on our previous experimental protocols (Pintori et al. 2022; Piva et al. 2018) and are shown in the figure legends. Due to experimental protocol criteria (e.g. acquisition criteria during lever press shaping phase), some animals were excluded from statistical analysis, thus reducing group sizes in few cases.

### Apparatus

Mice were trained and tested in operant chambers encased in sound-insulated cubicles equipped with ventilation fans (Med Associates Inc., Georgia Regional Industrial Park, Fairfax, VT, USA). Each chamber (Context A) was equipped with two levers, an active and an inactive lever symmetrically oriented laterally to the food magazine, on the frontal panel. Levers were located 2 cm above and food magazine at the same level of the grid floor. A 2-W white house light was located 10 cm above the grid floor on the back panel of the operant chambers and provided ambient illumination during the entire session duration of all the experimental phases, except for time-out (TO) periods during training and extinction phases. Context B was a modified version of the operant chamber, with 1-cm blank striped sheets on all the walls and a 1-cm side grid on the floor (Auber et al. 2014). Lever presses and pellet deliveries were recorded, as well as schedule parameters and data acquisition were controlled, by Med-PC IV software (Med Associates Inc., Georgia Regional Industrial Park, Fairfax, VT, USA). Contextual bias was controlled counterbalancing Contexts A and B for the experiments, with half of the mice of each experimental group conditioned in Context A and the other half conditioned in Context B.

### Lever press shaping and sucrose self-administration

Mice were initially trained to associate right lever presses with sucrose pellets as reinforcement in the conditioning context (sucrose-paired context, CxA). The schedule was FR1: 20-mg sucrose food pellet (Bilaney Consultants Ltd, UK), no TO, session duration up to 50 reinforcements or 4 h. Once the criterion of 50 reinforcements/session was reached, mice started training in the conditioning context. During training, right (active) lever presses corresponded to the delivery of sucrose reinforcement with the schedule: FR1: 20-mg sucrose pellet, 60-s TO, session duration up to 12 reinforcements or 1 h. During TO period, right lever presses had no programmed consequences. Light was ON throughout shaping and training sessions, except for TO periods during which it switched OFF. Left (inactive) lever presses were never associated with programmed consequences. Training lasted for 10 continuous days, and all lever presses during shaping and training were recorded.

### Lever extinction

Twenty-four hours after the last training session, mice started extinction training, receiving 30-min daily session of instrumental extinction in the extinction-paired context (CxB). Extinction session schedule was maintained identical to training schedule, except for a fixed duration (30 min) and for the absence of any delivery of sucrose pellets. The extinction phase lasted until mice performed, for three consecutive sessions, less than 50% of ALPs pressed at the first extinction session, or for a maximum of 7 consecutive days (Auber et al. 2014; Piva et al. 2020).

### Environmental treatment: acute Environmental Enrichment exposure

Twenty-four hours after the last extinction session, 2 groups of mice of each housing condition (2 social and 2 isolated groups) were exposed for 22 h to Environmental Enrichment (EE groups).

In the Exp. 1 (social condition), EE consisted in a rat two-level housing cage (each cage: 35.6 × 48.5 × 21.8 cm, Optirat Gen II, Animal Care Systems) where 3 or 4 mice from the same social home cage were housed with various objects (toys with different materials, shapes and colours, i.e. plastic balls and ladders, wood bricks), shelters and tunnels (Fig. 1A). In the Exp. 2 (isolated condition), EE consisted in a novel housing cage (12 × 17.5 × 35.5 cm, Sealsafe Plus GM 500, Tecniplast), where mice from isolation condition were single housed with various objects (toys with different materials, shapes and colours, i.e. plastic ball and ladder, wood brick) and tunnel (Fig. 1B). In both experiments, control NoEE groups were kept in their home cages. In the Exp. 1, home cage bias was controlled counterbalancing the subdivision of littermates in the two environmental manipulations, with half of the mice of each home cage assigned to EE and the other half assigned to NoEE group.

### Pharmacological treatment: acute fluoxetine administration

One hour before sucrose-seeking test, i.e. immediately after the end of EE exposure, 2 groups of mice (Fluox, EE-Fluox) were injected intraperitoneally with fluoxetine 10 mg/kg. Control groups (NoEE, EE) were injected with vehicle solution.

### Cx-induced sucrose-seeking test (Renewal effect)

One hour after pharmacological treatment, Cx-induced sucrose-seeking was tested in the sucrose-paired context (CxA). Test session lasted for 30 min, with house light ON throughout the session and no TO. Both levers were presented but not associated with programmed consequences.

### Statistical analysis

All the numerical data are given as mean ± SEM. Data were tested for normal distribution using Shapiro–Wilk’s test. In each experiment, ALPs and ILPs of training and extinction sessions were separately analysed for possible pre-existing group differences with a repeated-measures (RM) two-way analysis of variance (ANOVA) followed by Tukey’s multiple comparisons post hoc test for factors Session (mean of the last three S/A sessions, first extinction session, mean of the last three sessions of extinction phase) and Treatment (NoEE, EE, Fluox, EE-Fluox) or housing conditions (social, isolation). The same statistical analysis was used to assess the effect of the different treatments (NoEE, EE, Fluox, EE-Fluox) on Cx-induced sucrose seeking test (Session: last three sessions of extinction phase, test) within each living condition. Post hoc tests were conducted only when a significant main effect and/or interaction were detected. Differences were considered significant at *p* < 0.05. All analyses were performed using the GraphPad software package (Prism, version 8; GraphPad, San Diego, CA, USA).

## Results

### Experiment 1. Effects of brief EE exposure and fluoxetine combination on Cx-induced sucrose-seeking under social housing condition

**Fig. 2:**
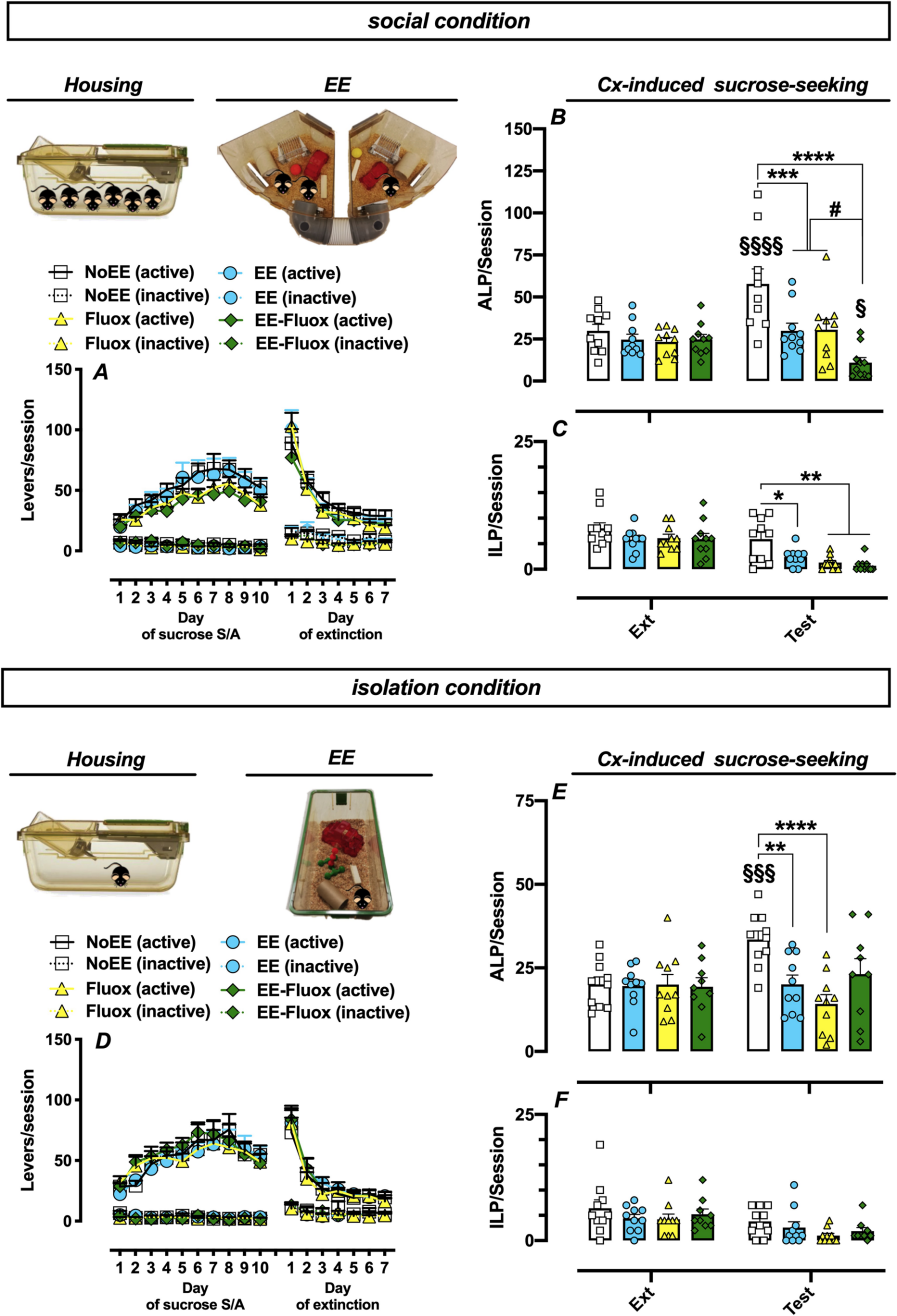
Effect of brief EE exposure and fluoxetine combination on Cx-induced sucrose-seeking in social and isolation living condition. **(A, D)** Active and inactive lever presses (ALPs, ILPs) during sucrose self-administration training (S/A) and extinction training in the extinction context (Ext). **(B, E)** Active and **(C, F)** inactive lever presses in the extinction context (mean last three extinction sessions) and in the sucrose-paired context during test. Data are presented as mean ± SEM. RM Two-way ANOVA, Tukey’s post hoc. $ = p < 0.05, $$$ = p < 0.001, $$$$ = p < 0.0001 vs. Ext; * = p < 0.05 ** = p < 0.01*** = p < 0.001 **** = p < 0.0001 vs. NoEE group; # = p < 0.05 vs. EE or Fluox groups. Social condition: n = 10 mice per group; isolation condition: NoEE, EE, EE-Fluox n = 10 per group, Fluox n = 9

In order to assess the effect of single and combined treatments on Cx-induced sucrose-seeking under social housing condition, we exposed mice to (i) brief EE exposure, or (ii) acute fluoxetine (10 mg/kg, i.p.) administration, or (iii) EE-fluoxetine combination before final test. No significant differences in lever presses among groups were observed during sucrose self-administration and lever extinction phase (Fig. 2A). As shown in Fig. 2B, brief EE exposure and acute fluoxetine administration inhibited Cx-induced sucrose-seeking. Interestingly, their combination induced a more pronounced reduction of Cx-induced sucrose-seeking behaviours.

Two-way ANOVA analysis of ALPs showed a main effect of session [*F*_(1,36)_ = 6.53; *p* < 0.05], of treatment [*F*_(3,36)_ = 6.97; *p* < 0.001] and of interaction [*F*_(3,36)_ = 10.74; *p* < 0.0001]. Tukey’s post hoc test revealed a significant increase of ALPs in the NoEE group when re-exposed to sucrose-paired context as compared to extinction context and to the other groups (EE, Fluox, EE-Fluox). Moreover, Tukey’s post hoc test revealed a significant decrease of ALPs in the EE-Fluox group when re-exposed to sucrose-paired context as compared to extinction context and to EE and Fluox groups (Fig. 2B).

Two-way ANOVA analysis of ILPs showed a main effect of session [*F*_(1,36)_ = 34.73; *p* < 0.0001], of treatment [*F*_(3,36)_ = 8.53; *p* < 0.001], but not of interaction. Tukey’s post hoc test revealed a significant decrease of ILPs in EE, Fluox and EE-Fluox groups as compared to the NoEE group when re-exposed to sucrose-paired context (Fig. 2C).

These findings suggest that, under social condition, the association between brief EE exposure and fluoxetine potentiated the inhibition of Cx-induced sucrose-seeking behaviours compared to single treatments alone.

### Experiment 2. Effects of brief EE exposure and fluoxetine combination on Cx-induced sucrose-seeking under isolated housing condition

In order to assess the effect of single and combined treatments on Cx-induced sucrose-seeking under isolation housing condition, we exposed mice to (i) brief EE exposure, or (ii) acute fluoxetine (10 mg/kg, i.p.) administration, or (ii) EE-fluoxetine combination before final test. No significant differences in lever presses among groups were observed during sucrose self-administration and lever extinction phase (Fig. 2D). As shown in Fig. 2E, although either brief EE exposure or fluoxetine inhibited Cx-induced sucrose-seeking, their combination did not potentiate this inhibition.

Two-way ANOVA analysis of ALPs showed a main effect of treatment [*F*_(3,35)_ = 3.02; *p* < 0.05], of interaction [*F*_(3,35)_ = 6.25; *p* < 0.005], but not of session. Tukey’s post hoc test revealed a significant increase of ALPs in NoEE group when re-exposed to sucrose-paired context as compared to extinction context and to EE and Fluox groups (Fig. 2E). Differently, EE-Fluox group exhibited no changes of ALPs when re-exposed to sucrose-paired context as compared to the other groups (NoEE, EE, Fluox) and to extinction context (Fig. 2E). No statistical differences of ILPs among groups were observed (Fig. 2F).

Thus, under isolated conditions, brief EE exposure and fluoxetine combination did not potentiate the inhibition of Cx-induced sucrose-seeking behaviours.

## Discussion

In the present study, we aimed to explore the possible combination of brief EE exposure and fluoxetine effects in inhibiting sucrose-seeking behaviours, and whether living environment affected treatment’s efficacy.

We showed that brief EE exposure and fluoxetine combination potentiates the inhibition of context-induced sucrose-seeking in social but not in isolation housing condition. Therefore, our data demonstrated that the positive interaction between pharmacological (fluoxetine) and non-pharmacological (EE) interventions on preventing relapse is dependent on living environmental conditions.

The discriminative role of living conditions, in our study social and isolation housing, is consistent with the literature pointing out the quality of living environment as a critical intervening factor determining treatment efficacy in neuropsychiatric diseases, such as depression (see review (Branchi and Giuliani 2021)). Although a role of living conditions in the vulnerability to addiction has been shown (Ajonijebu et al. 2017; Caprioli et al. 2007), little is known about their impact on the therapeutic efficacy to prevent relapse (Liu et al. 2019; Polcin et al. 2010). In this study, we demonstrated that a social living environment favours a positive interaction (synergism or additive effect) between EE and fluoxetine in inhibiting sucrose relapse in adult mice. To the best of our knowledge, we are the first reporting a potentiation of beneficial effects induced by EE and fluoxetine (SSRI drug) association in an animal model of relapse, as well as the role of living environment.

It is reasonable to hypothesize that the lack of potentiation observed in isolated mice, could be due to neurobiological changes induced by the different housing conditions. It is well known that isolated rodents display different neurochemical and behavioural profiles compared to social living animals (see review (Hall 1998)). For instance, isolated animals exhibit a behavioural pattern called *‘social isolation syndrome’*, including higher hyperactivity (Brenes et al. 2008), impulsivity (Perry et al. 2008) and anxiety (Hall et al. 1998; Weiss et al. 2004). Moreover, it has been reported increased 5-HT turnover in the nucleus accumbens (NAc) and in the prefrontal cortex (PFC) of isolated rats, which was correlated with depressive-like behaviours (Brenes et al. 2008). Finally, isolation alters the mesolimbic dopaminergic system and the response to several substances of abuse (Hall 1998; Robbins et al. 1996), increasing drug-seeking and -intake (Wolffgramm and Heyne 1991). Consistently, our isolated mice exhibited greater sucrose-seeking behaviours during sucrose self-administration as compared to social mice (data not shown). Noteworthy, in our experimental conditions, social and isolated mice reached the same number of rewards during each training session (12 sugar pellets). Therefore, the higher responding exhibited by isolated mice may lead to differences in occasion-setting properties of sucrose-paired context rather than in action-outcome associations, that in turn may influence the response at renewal test.

Although social deprivation has a strong impact especially during the early life period, isolation induces marked neuroplasticity alterations regardless of timing of exposure (Begni et al. 2020). In fact, it has been demonstrated that social deprivation at adulthood reduces BDNF and Arc mRNA levels in the PFC, together with a hyperactive phenotype similar to that observed in rats isolated during adolescence (Begni et al. 2020). Consistently, Miura and colleagues (Miura et al. 2002) showed that isolation during adulthood induces changes of mesocorticolimbic systems activity, in particular attenuating dopamine and 5-HT response to stress. Therefore, it is plausible that these changes induced by social deprivation during adulthood counteract the possible combination between brief EE exposure and fluoxetine effects in inhibiting context-induced sucrose-seeking. In line with this speculation, it has been demonstrated a reciprocal interaction and influence between BDNF and 5-HT pathways (Hayley et al. 2005), which are the major targets of EE and fluoxetine.

However, since the complexity of our behavioural paradigms and the three factors involved (EE, fluoxetine, living conditions), we cannot exclude that the behavioural outcomes observed might be the result of a complex interplay between other molecular mechanisms (Begni et al. 2020; Eckert and Abraham 2013; Solinas et al. 2021; van Praag et al. 2000; Zorzin et al. 2021). For instance, the different *‘EE experience’* due to the specific EE complexity and features used in social and isolated mice (2-level cage with littermates vs. new standard home cage without social component) may also represent another factor that could affect the efficacy of EE-fluoxetine combination in inhibiting sucrose-seeking. In this view, our results may represent an additive effect between EE and fluoxetine treatment rather than a synergism, due to an *EE dose-dependent* effect on the same mechanism.

Moreover, in our experimental conditions, acute fluoxetine (10 mg/kg) administration inhibited Cx-induced sucrose-seeking in both living conditions, as demonstrated by no increase of lever pressing in social and isolated fluoxetine groups when re-exposed to sucrose-paired context. These data are consistent with the literature on acute (Burmeister et al. 2003; Simon O’Brien et al. 2011) and chronic (Baker et al. 2001) fluoxetine treatments in animals models of addiction. For instance, Burmeister and colleagues (Burmeister et al. 2003) showed that acute fluoxetine administration (10 mg/kg) reduced cue-induced cocaine-seeking with or without cocaine priming in isolated rats. Similarly, fluoxetine (10 mg/kg) completely blocked ethanol self-administration in both dependent and non-dependent group-housed rats (Simon O’Brien et al. 2011). Importantly, both studies excluded motor impairments induced by fluoxetine as possible confounding factor. The authors suggested that the reduction of drug-seeking behaviours may be due to a decrease of incentive motivational value of drug-associated stimuli, most likely mediated via enhancement of 5-HT neurotransmission (Burmeister et al. 2003; Simon O’Brien et al. 2011). Consistent with this hypothesis, 5-HT depletion potentiated sucrose-seeking behaviours (Fletcher et al. 1999; Tran-Nguyen et al. 2001), as well as increased break-point on progressive ratio schedules of food reinforcement (Roberts et al. 1994). Although the decrease of both active and inactive lever responding observed in social fluoxetine mice might suggest a general reduction of motor activity, a similar decrease has also been observed in social EE mice, while isolated mice exhibited only a downward trend. Therefore, it is plausible that also in our experimental paradigms, fluoxetine attenuates sucrose-seeking behaviours, most likely reducing incentive motivational value of sucrose-associated context.

Neither preclinical nor clinical studies evaluated the impact of social living background on SSRIs efficacy to inhibit relapse. As suggested above for the EE-fluoxetine combination, the pre-existing neurochemical substrates induced by the different housing conditions may explain the different sensitivity to fluoxetine observed in our study, but also the inconsistent and contradictory clinical results observed with fluoxetine treatment on addiction disorders (Balter et al. 2014; Benowitz 2008). Other reasons may be related to the treatment features (e.g. low vs. high doses, chronic vs. acute) used across studies. As a matter of fact, the lack of clinical efficacy of SSRIs has been observed with low doses used to treat depression, whereas clinical utility has been observed with high doses successfully used to treat obsessive–compulsive disorders (Covi et al. 1995; Moeller et al. 2007), which are comparable to the dose used in the present study (10 mg/kg). Moreover, several clinical trials involving chronic SSRI treatment failed to show efficacy in addiction disorders due to the compliance necessary to continue a withdrawal period (Batki et al. 1996; Grabowski et al. 1995). Therefore, besides living conditions, a single high dose treatment such as those used in our and other preclinical studies (Burmeister et al. 2003; Simon O’Brien et al. 2011), may represent the optimal solution to increase clinical SSRIs efficacy in treating addictive behaviours.

On the other hand, brief EE exposure inhibited context-induced sucrose-seeking regardless of living conditions. Although social interaction may consistently impact reward seeking and taking (Brenes et al. 2008; Gill and Cain 2011; Thiel et al. 2010), brief EE exposure attenuates sucrose-seeking with the same magnitude in social and isolated mice. These results are consistent with EE literature on drug/food-taking and -seeking (Grimm et al. 2008; Solinas et al. 2010), confirming the *‘curative’* effects of EE on addictive behaviours, even as a brief (22 h) single intervention. According to Grimm (Grimm et al. 2013), our data confirm that also an acute exposure to EE without social and motor components, as applied on our isolated mice, is able to reduce sucrose-seeking. Therefore, our results demonstrated that brief EE exposure can inhibit renewal regardless of its complexity and social environment. A possible explanation is that a *‘low EE dose’* is enough to inhibit sucrose-seeking but is not sufficient to potentiate the inhibition when combined with fluoxetine, consistent with an additive effect hypothesis. Nevertheless, EE may inhibit sucrose-seeking acting on different neuronal pathways and/or with different mechanisms compared to fluoxetine (supporting synergistic effect), which are not affected by living environment.

Taken together, our results demonstrated that living environment influences either single or combined therapeutic interventions for addiction disorders. In particular, we demonstrated that brief EE exposure and fluoxetine combination potentiates the inhibition of context-induced sucrose-seeking only in social condition. This differential behavioural sensitivity to combined treatments may be related to the neurochemical-molecular changes modulated by the living environment.

However, the study owns some limitations. First, our study was focused on the behavioural effects induced by the single and combined treatments in different living environment conditions. Therefore, ad-hoc molecular studies are needed on the underlying neurochemical changes induced by the different living environments, as well as on those underlying the EE-fluoxetine combination, to characterize their interaction. In addition, the inhibitory effect of brief EE exposure observed in this study is in contrast with the potentiation of context-induced sucrose-seeking that we recently observed in rats using the same experimental paradigm (Pintori et al. 2022). Consistently, some important rats-mice differences have been reported in the effects of EE on sensitivity to drugs of abuse (i.e. cocaine, amphetamine) (Bardo et al. 1999; Bowling and Bardo 1994; Solinas et al. 2009). Therefore, possible interspecies differences on the efficacy of fluoxetine as single or combined treatment cannot be excluded. Finally, experiments are needed to extent and confirm the beneficial effects of EE-fluoxetine combination on drug addiction.

In conclusion, our study suggests that social living condition might influence the therapeutic efficacy of single and integrative approaches for treating addiction. In fact, living environment influenced the efficacy of EE/fluoxetine interaction in inhibiting relapse to sucrose. This view may be helpful to better understand the effects, as well as facilitate the clinical application of brief environment exposure and enviromimetic treatment through the control of living environmental conditions in patients. In humans, this could be achieved by exposing patients concomitantly to social conditions, for instance through group psychotherapy, or applying these interventions in residential communities. Indeed, setting up proper living conditions (e.g. high social economic status or positive family and peer relations in humans) to boost the efficacy of different therapeutic approaches may represent a fundamental strategy to treat addiction disorders.
